# Supplementary material for: Single Cell Transcriptomic and Chromatin Profiles Suggest Layer Vb Is the Only Layer With Shared Excitatory Cell Types in the Medial and Lateral Entorhinal Cortex
Source: Front Neural Circuits. 2022 Jan 26;15:806154. doi: 10.3389/fncir.2021.806154 (PMC8826650; doi:10.3389/fncir.2021.806154)
Supplement: Supplementary file 14 [file Data_Sheet_1.ZIP › Sup_vs_Deep_Motifs/knownResults.html]

Sup\_vs\_Deep\_Motifs/ - Homer Known Motif Enrichment Results


# Homer Known Motif Enrichment Results (Sup\_vs\_Deep\_Motifs/)

Homer *de novo* Motif Results  
Gene Ontology Enrichment Results  
Known Motif Enrichment Results (txt file)  
Total Target Sequences = 33506, Total Background Sequences = 8535

|  |  |  |  |  |  |  |  |  |  |  |  |
| --- | --- | --- | --- | --- | --- | --- | --- | --- | --- | --- | --- |
| Rank | Motif | Name | P-value | log P-pvalue | q-value (Benjamini) | # Target Sequences with Motif | % of Targets Sequences with Motif | # Background Sequences with Motif | % of Background Sequences with Motif | Motif File | SVG |
| 1 | A T G C T G C A A G T C C G T A A G T C A G T C A C G T A C T G A T G C G T C A | E2A(bHLH),near\_PU.1/Bcell-PU.1-ChIP-Seq(GSE21512)/Homer | 1e-2677 | -6.165e+03 | 0.0000 | 17249.0 | 51.48% | 2000.7 | 23.44% | motif file (matrix) | svg |
| 2 | A G C T T C G A G T A C T C G A A T G C A T G C G C A T A T C G A G T C A G C T | Snail1(Zf)/LS174T-SNAIL1.HA-ChIP-Seq(GSE127183)/Homer | 1e-2367 | -5.450e+03 | 0.0000 | 12621.0 | 37.66% | 1238.2 | 14.50% | motif file (matrix) | svg |
| 3 | T A C G C T A G T A C G A G T C C G T A A G T C A G T C A C G T A C T G A G T C G A T C T A G C | Slug(Zf)/Mesoderm-Snai2-ChIP-Seq(GSE61475)/Homer | 1e-2053 | -4.729e+03 | 0.0000 | 9119.0 | 27.21% | 759.0 | 8.89% | motif file (matrix) | svg |
| 4 | T G C A G T A C C G T A A T C G A C T G A C G T C T A G C G A T T C G A A G T C | ZEB1(Zf)/PDAC-ZEB1-ChIP-Seq(GSE64557)/Homer | 1e-1835 | -4.225e+03 | 0.0000 | 18289.0 | 54.58% | 2592.4 | 30.37% | motif file (matrix) | svg |
| 5 | C G T A T G A C T C G A A G T C C G T A A T C G A T G C A C G T A C T G A G T C | E2A(bHLH)/proBcell-E2A-ChIP-Seq(GSE21978)/Homer | 1e-1695 | -3.903e+03 | 0.0000 | 17380.0 | 51.87% | 2461.3 | 28.83% | motif file (matrix) | svg |
| 6 | C T A G A C T G T G C A A G T C C G T A A C T G A C T G A C G T C T A G C G A T T A C G A G T C | ZEB2(Zf)/SNU398-ZEB2-ChIP-Seq(GSE103048)/Homer | 1e-1427 | -3.288e+03 | 0.0000 | 11418.0 | 34.07% | 1365.3 | 15.99% | motif file (matrix) | svg |
| 7 | T G A C A T G C C G T A A T C G A T G C C A G T C A T G A C T G A G T C G T A C | HEB(bHLH)/mES-Heb-ChIP-Seq(GSE53233)/Homer | 1e-1408 | -3.243e+03 | 0.0000 | 20769.0 | 61.98% | 3427.6 | 40.15% | motif file (matrix) | svg |
| 8 | T A C G T A C G C T A G T C A G A G T C C G T A A T C G A T G C A C G T A C T G A G T C G A C T | Ascl2(bHLH)/ESC-Ascl2-ChIP-Seq(GSE97712)/Homer | 1e-1263 | -2.909e+03 | 0.0000 | 14143.0 | 42.21% | 1993.2 | 23.35% | motif file (matrix) | svg |
| 9 | T C G A A G T C C G T A A T C G T A G C A C G T A C T G A G C T A C G T A G T C | Ptf1a(bHLH)/Panc1-Ptf1a-ChIP-Seq(GSE47459)/Homer | 1e-1100 | -2.534e+03 | 0.0000 | 25041.0 | 74.73% | 4780.2 | 56.00% | motif file (matrix) | svg |
| 10 | C T A G A G T C T A C G T A C G T G A C C G T A A C T G T A G C G C A T C A T G A T G C A G C T | Ascl1(bHLH)/NeuralTubes-Ascl1-ChIP-Seq(GSE55840)/Homer | 1e-855 | -1.970e+03 | 0.0000 | 17887.0 | 53.38% | 3118.5 | 36.53% | motif file (matrix) | svg |
| 11 | T C A G T G A C G T A C C G T A A C G T T G A C A C G T T C A G A G C T G A C T | NeuroD1(bHLH)/Islet-NeuroD1-ChIP-Seq(GSE30298)/Homer | 1e-313 | -7.222e+02 | 0.0000 | 12174.0 | 36.33% | 2293.1 | 26.86% | motif file (matrix) | svg |
| 12 | G C A T G C A T C T G A A C G T C T G A A C G T C G T A C G T A C G T A A G T C G T C A G T C A | Foxf1(Forkhead)/Lung-Foxf1-ChIP-Seq(GSE77951)/Homer | 1e-284 | -6.542e+02 | 0.0000 | 11776.0 | 35.14% | 2237.7 | 26.21% | motif file (matrix) | svg |
| 13 | T C G A T G A C G T A C C G T A C A G T T G A C A C G T A C T G A G C T A G C T | NeuroG2(bHLH)/Fibroblast-NeuroG2-ChIP-Seq(GSE75910)/Homer | 1e-254 | -5.870e+02 | 0.0000 | 18985.0 | 56.66% | 4041.4 | 47.34% | motif file (matrix) | svg |
| 14 | T C A G A C G T T C G A T A G C A G T C C G T A A C T G G T A C A C G T A C T G A T C G A G T C | Atoh1(bHLH)/Cerebellum-Atoh1-ChIP-Seq(GSE22111)/Homer | 1e-248 | -5.717e+02 | 0.0000 | 14670.0 | 43.78% | 2976.9 | 34.87% | motif file (matrix) | svg |
| 15 | C G T A G C T A C G A T C T A G A C G T G T C A C G T A C G T A A G T C C G T A T G C A T A C G | FoxL2(Forkhead)/Ovary-FoxL2-ChIP-Seq(GSE60858)/Homer | 1e-246 | -5.676e+02 | 0.0000 | 10666.0 | 31.83% | 2028.1 | 23.76% | motif file (matrix) | svg |
| 16 | T C G A A G T C C G T A A T C G A T G C C G A T A C T G A G T C A G C T A C T G | Tcf12(bHLH)/GM12878-Tcf12-ChIP-Seq(GSE32465)/Homer | 1e-243 | -5.608e+02 | 0.0000 | 10411.0 | 31.07% | 1973.7 | 23.12% | motif file (matrix) | svg |
| 17 | A G C T T G A C C G A T C G A T C T A G A C G T C A G T C A G T G C T A A G T C | FOXK1(Forkhead)/HEK293-FOXK1-ChIP-Seq(GSE51673)/Homer | 1e-236 | -5.435e+02 | 0.0000 | 11877.0 | 35.44% | 2325.1 | 27.24% | motif file (matrix) | svg |
| 18 | T C G A T C G A A G T C C G T A C T A G T A G C A C G T A C T G | MyoG(bHLH)/C2C12-MyoG-ChIP-Seq(GSE36024)/Homer | 1e-234 | -5.409e+02 | 0.0000 | 11308.0 | 33.75% | 2193.2 | 25.69% | motif file (matrix) | svg |
| 19 | A C T G C A T G T C G A A C G T A C T G C G T A A T C G A C G T G T A C C G T A G A C T A G T C | Fos(bZIP)/TSC-Fos-ChIP-Seq(GSE110950)/Homer | 1e-234 | -5.389e+02 | 0.0000 | 7950.0 | 23.72% | 1428.4 | 16.73% | motif file (matrix) | svg |
| 20 | C T G A T G A C T G A C C G T A A C G T T G A C A G C T C T A G A C G T G A C T | Olig2(bHLH)/Neuron-Olig2-ChIP-Seq(GSE30882)/Homer | 1e-233 | -5.369e+02 | 0.0000 | 21526.0 | 64.24% | 4734.1 | 55.45% | motif file (matrix) | svg |
| 21 | A C T G C T A G T C G A C G A T C A T G G C T A A T C G C G A T G T A C G C T A A G C T G T A C | Fra1(bZIP)/BT549-Fra1-ChIP-Seq(GSE46166)/Homer | 1e-229 | -5.274e+02 | 0.0000 | 7571.0 | 22.59% | 1350.1 | 15.81% | motif file (matrix) | svg |
| 22 | C T A G T C G A C G A T A C T G C G T A T A C G A G C T T G A C G C T A A C G T G A T C T A G C | Fosl2(bZIP)/3T3L1-Fosl2-ChIP-Seq(GSE56872)/Homer | 1e-224 | -5.176e+02 | 0.0000 | 4624.0 | 13.80% | 727.8 | 8.52% | motif file (matrix) | svg |
| 23 | T G A C A G T C C G T A A C T G G T A C A C G T A C T G A C G T G A C T G A T C | Twist2(bHLH)/Myoblast-Twist2.Ty1-ChIP-Seq(GSE127998)/Homer | 1e-222 | -5.118e+02 | 0.0000 | 19931.0 | 59.48% | 4337.5 | 50.81% | motif file (matrix) | svg |
| 24 | C T A G T C G A T G A C A G T C C G T A A C T G G T A C A C G T A C T G A C T G | BHLHA15(bHLH)/NIH3T3-BHLHB8.HA-ChIP-Seq(GSE119782)/Homer | 1e-221 | -5.106e+02 | 0.0000 | 17391.0 | 51.90% | 3691.6 | 43.24% | motif file (matrix) | svg |
| 25 | C T A G T C G A A C G T A C T G C G T A T A G C C G A T G T A C C G T A A G C T G A T C G T A C | Jun-AP1(bZIP)/K562-cJun-ChIP-Seq(GSE31477)/Homer | 1e-216 | -4.978e+02 | 0.0000 | 3557.0 | 10.62% | 521.5 | 6.11% | motif file (matrix) | svg |
| 26 | C G A T C T A G A C G T G T C A C G T A C G T A A G T C C G T A | Foxo3(Forkhead)/U2OS-Foxo3-ChIP-Seq(E-MTAB-2701)/Homer | 1e-213 | -4.915e+02 | 0.0000 | 9077.0 | 27.09% | 1708.0 | 20.01% | motif file (matrix) | svg |
| 27 | C T A G T C G A G C A T C A T G G C T A T A G C C G A T G T A C C T G A A G C T | JunB(bZIP)/DendriticCells-Junb-ChIP-Seq(GSE36099)/Homer | 1e-207 | -4.768e+02 | 0.0000 | 7273.0 | 21.70% | 1310.3 | 15.35% | motif file (matrix) | svg |
| 28 | C T A G T C G A A C G T A C T G C G T A A T G C A C G T G T A C C G T A A G C T G A T C G T A C | Atf3(bZIP)/GBM-ATF3-ChIP-Seq(GSE33912)/Homer | 1e-202 | -4.654e+02 | 0.0000 | 8496.0 | 25.35% | 1590.7 | 18.63% | motif file (matrix) | svg |
| 29 | C A T G C T A G T C G A A C G T A C T G C G T A T A G C C G A T T G A C C G T A A G C T G A T C | Fra2(bZIP)/Striatum-Fra2-ChIP-Seq(GSE43429)/Homer | 1e-199 | -4.591e+02 | 0.0000 | 6476.0 | 19.33% | 1144.3 | 13.40% | motif file (matrix) | svg |
| 30 | A C G T T C G A T C G A A G T C G T C A T A C G A T G C A C G T A C T G A G C T | Myf5(bHLH)/GM-Myf5-ChIP-Seq(GSE24852)/Homer | 1e-189 | -4.355e+02 | 0.0000 | 8450.0 | 25.22% | 1597.1 | 18.71% | motif file (matrix) | svg |
| 31 | C A G T T G C A A C G T A C T G C G T A A T C G C G A T T G A C C G T A A C G T | BATF(bZIP)/Th17-BATF-ChIP-Seq(GSE39756)/Homer | 1e-188 | -4.337e+02 | 0.0000 | 8454.0 | 25.23% | 1599.7 | 18.74% | motif file (matrix) | svg |
| 32 | A G C T A G T C A G T C A C G T C T A G A C G T A C G T A C G T C G T A A G T C G A T C C G T A | FOXP1(Forkhead)/H9-FOXP1-ChIP-Seq(GSE31006)/Homer | 1e-185 | -4.267e+02 | 0.0000 | 5436.0 | 16.22% | 935.2 | 10.96% | motif file (matrix) | svg |
| 33 | T C G A C A G T A C T G A G C T C G T A C G T A A C G T A C G T C T G A T A G C | Dlx3(Homeobox)/Kerainocytes-Dlx3-ChIP-Seq(GSE89884)/Homer | 1e-183 | -4.226e+02 | 0.0000 | 9106.0 | 27.17% | 1755.6 | 20.57% | motif file (matrix) | svg |
| 34 | T A C G T G C A A G T C C G T A A C G T T G A C A C G T A C T G A C T G G C A T | TCF4(bHLH)/SHSY5Y-TCF4-ChIP-Seq(GSE96915)/Homer | 1e-180 | -4.163e+02 | 0.0000 | 18285.0 | 54.57% | 3989.2 | 46.73% | motif file (matrix) | svg |
| 35 | G C T A T C G A C G T A C T A G A G C T G T C A G T C A C G T A A G T C C G T A | FOXA1(Forkhead)/MCF7-FOXA1-ChIP-Seq(GSE26831)/Homer | 1e-179 | -4.122e+02 | 0.0000 | 11810.0 | 35.24% | 2396.5 | 28.07% | motif file (matrix) | svg |
| 36 | A T G C T A C G A G C T T G C A C G T A C G A T A C G T C T G A | DLX5(Homeobox)/BasalGanglia-Dlx5-ChIP-seq(GSE124936)/Homer | 1e-175 | -4.032e+02 | 0.0000 | 10073.0 | 30.06% | 1992.7 | 23.34% | motif file (matrix) | svg |
| 37 | T C G A A C G T C A T G G C T A T A G C C G A T G T A C G C T A A C G T A T G C | AP-1(bZIP)/ThioMac-PU.1-ChIP-Seq(GSE21512)/Homer | 1e-174 | -4.023e+02 | 0.0000 | 9063.0 | 27.05% | 1758.9 | 20.60% | motif file (matrix) | svg |
| 38 | A G C T G T C A C G T A A C G T A C G T C T G A T C A G A T G C | Lhx2(Homeobox)/HFSC-Lhx2-ChIP-Seq(GSE48068)/Homer | 1e-174 | -4.021e+02 | 0.0000 | 12684.0 | 37.85% | 2613.5 | 30.61% | motif file (matrix) | svg |
| 39 | G C T A T C G A C G T A C T A G A G C T G T C A G T C A C G T A A G T C C G T A | FOXA1(Forkhead)/LNCAP-FOXA1-ChIP-Seq(GSE27824)/Homer | 1e-156 | -3.603e+02 | 0.0000 | 13810.0 | 41.21% | 2919.5 | 34.20% | motif file (matrix) | svg |
| 40 | C G A T T G C A T G C A G A T C C G T A A C T G T G A C G A C T C A T G A C T G | Tcf21(bHLH)/ArterySmoothMuscle-Tcf21-ChIP-Seq(GSE61369)/Homer | 1e-135 | -3.115e+02 | 0.0000 | 10890.0 | 32.50% | 2253.3 | 26.39% | motif file (matrix) | svg |
| 41 | C G T A C A T G A G T C G A C T T G C A C G T A A C G T A C G T C T G A T C A G | Lhx1(Homeobox)/EmbryoCarcinoma-Lhx1-ChIP-Seq(GSE70957)/Homer | 1e-132 | -3.044e+02 | 0.0000 | 13154.0 | 39.26% | 2806.8 | 32.88% | motif file (matrix) | svg |
| 42 | A T C G A T G C A G T C T A G C G A C T T C G A G C T A G C A T A G C T C T G A | DLX1(Homeobox)/BasalGanglia-Dlx1-ChIP-seq(GSE124936)/Homer | 1e-116 | -2.688e+02 | 0.0000 | 14621.0 | 43.63% | 3200.5 | 37.49% | motif file (matrix) | svg |
| 43 | T A C G T A G C G C T A C G A T C T A G A C G T C A G T C A G T G C T A A G T C G T C A G C A T | FOXK2(Forkhead)/U2OS-FOXK2-ChIP-Seq(E-MTAB-2204)/Homer | 1e-112 | -2.596e+02 | 0.0000 | 7287.0 | 21.75% | 1447.4 | 16.95% | motif file (matrix) | svg |
| 44 | A T G C T A C G A G T C G A C T T G C A C T G A G A C T A C G T C T G A T C A G | LHX9(Homeobox)/Hct116-LHX9.V5-ChIP-Seq(GSE116822)/Homer | 1e-107 | -2.483e+02 | 0.0000 | 15066.0 | 44.96% | 3331.9 | 39.03% | motif file (matrix) | svg |
| 45 | A T C G A T C G A T G C G A C T T C G A C G T A G C A T A G C T C T G A T A C G | DLX2(Homeobox)/BasalGanglia-Dlx2-ChIP-seq(GSE124936)/Homer | 1e-107 | -2.473e+02 | 0.0000 | 16157.0 | 48.22% | 3605.0 | 42.23% | motif file (matrix) | svg |
| 46 | C G A T T A C G T G A C G A C T C A T G C G T A T A C G A C G T G T A C C T G A | Bach2(bZIP)/OCILy7-Bach2-ChIP-Seq(GSE44420)/Homer | 1e-106 | -2.443e+02 | 0.0000 | 2458.0 | 7.34% | 394.5 | 4.62% | motif file (matrix) | svg |
| 47 | A T G C G A C T A C G T C T A G A C G T A C G T A C G T C T G A G A T C G C T A A G C T C G T A | Foxa2(Forkhead)/Liver-Foxa2-ChIP-Seq(GSE25694)/Homer | 1e-104 | -2.417e+02 | 0.0000 | 9186.0 | 27.41% | 1906.2 | 22.33% | motif file (matrix) | svg |
| 48 | T C A G C T A G A T G C G A C T T G C A C G T A A G C T A C G T C T G A T A C G | En1(Homeobox)/SUM149-EN1-ChIP-Seq(GSE120957)/Homer | 1e-94 | -2.185e+02 | 0.0000 | 17928.0 | 53.50% | 4084.4 | 47.84% | motif file (matrix) | svg |
| 49 | C G A T C T A G A C G T A C G T A C G T C G T A A G C T C G A T A G C T C G T A C T A G T A G C | FoxD3(forkhead)/ZebrafishEmbryo-Foxd3.biotin-ChIP-seq(GSE106676)/Homer | 1e-94 | -2.178e+02 | 0.0000 | 10379.0 | 30.97% | 2213.2 | 25.93% | motif file (matrix) | svg |
| 50 | A C T G A G T C G T C A C G T A A G T C C G T A C T A G C T A G G A C T C A T G | SCRT1(Zf)/HEK293-SCRT1.eGFP-ChIP-Seq(Encode)/Homer | 1e-88 | -2.038e+02 | 0.0000 | 2959.0 | 8.83% | 517.5 | 6.06% | motif file (matrix) | svg |
| 51 | A G T C T A G C G A C T A C G T C T A G A C G T A C G T A C G T C T G A A G T C G C T A G A C T C G T A C T A G A C T G | Foxa3(Forkhead)/Liver-Foxa3-ChIP-Seq(GSE77670)/Homer | 1e-81 | -1.869e+02 | 0.0000 | 4061.0 | 12.12% | 767.6 | 8.99% | motif file (matrix) | svg |
| 52 | A C G T C T A G A G C T A C G T A C G T C T G A A G T C G A C T A G C T C G T A | FOXM1(Forkhead)/MCF7-FOXM1-ChIP-Seq(GSE72977)/Homer | 1e-80 | -1.846e+02 | 0.0000 | 11757.0 | 35.09% | 2582.3 | 30.25% | motif file (matrix) | svg |
| 53 | C A T G A G T C G A C T C G T A C G A T G C A T G A C T G C A T C G A T C T A G C A T G T G A C | Mef2b(MADS)/HEK293-Mef2b.V5-ChIP-Seq(GSE67450)/Homer | 1e-79 | -1.820e+02 | 0.0000 | 12759.0 | 38.08% | 2831.3 | 33.17% | motif file (matrix) | svg |
| 54 | G T C A T G C A G C T A A G T C C G T A A C T G T G A C G C A T T C A G C A G T | Ap4(bHLH)/AML-Tfap4-ChIP-Seq(GSE45738)/Homer | 1e-74 | -1.726e+02 | 0.0000 | 12378.0 | 36.94% | 2748.6 | 32.20% | motif file (matrix) | svg |
| 55 | C G T A C G T A A G C T G A C T T G C A G T C A A C G T A G C T C T G A T C A G | Lhx3(Homeobox)/Neuron-Lhx3-ChIP-Seq(GSE31456)/Homer | 1e-67 | -1.551e+02 | 0.0000 | 16718.0 | 49.89% | 3854.6 | 45.15% | motif file (matrix) | svg |
| 56 | A T G C A G T C G A T C C G T A A C G T A C G T A C T G A C G T A G C T G A T C | Sox2(HMG)/mES-Sox2-ChIP-Seq(GSE11431)/Homer | 1e-66 | -1.542e+02 | 0.0000 | 8176.0 | 24.40% | 1749.6 | 20.50% | motif file (matrix) | svg |
| 57 | C T G A T C A G G T A C G C T A A C T G T G A C G C A T C A T G | SCL(bHLH)/HPC7-Scl-ChIP-Seq(GSE13511)/Homer | 1e-65 | -1.519e+02 | 0.0000 | 28567.0 | 85.25% | 6976.0 | 81.72% | motif file (matrix) | svg |
| 58 | C A T G G T A C G A C T G C T A C G T A C G T A C G T A G C T A G A C T C T G A T C A G G T A C | Mef2c(MADS)/GM12878-Mef2c-ChIP-Seq(GSE32465)/Homer | 1e-65 | -1.505e+02 | 0.0000 | 8214.0 | 24.51% | 1762.0 | 20.64% | motif file (matrix) | svg |
| 59 | A G T C A C G T A C T G A G C T A C G T A C G T G T C A A G T C | Foxo1(Forkhead)/RAW-Foxo1-ChIP-Seq(Fan\_et\_al.)/Homer | 1e-62 | -1.432e+02 | 0.0000 | 17565.0 | 52.42% | 4085.7 | 47.86% | motif file (matrix) | svg |
| 60 | C T G A T C A G A G T C C G T A A T C G A T G C C G A T A C T G A G T C G A C T A T C G A G T C | MyoD(bHLH)/Myotube-MyoD-ChIP-Seq(GSE21614)/Homer | 1e-61 | -1.425e+02 | 0.0000 | 7768.0 | 23.18% | 1664.2 | 19.49% | motif file (matrix) | svg |
| 61 | A T G C G T A C C G T A A G C T G C A T T A C G A G C T A G C T A G T C A G C T | Sox6(HMG)/Myotubes-Sox6-ChIP-Seq(GSE32627)/Homer | 1e-60 | -1.395e+02 | 0.0000 | 14689.0 | 43.84% | 3364.7 | 39.41% | motif file (matrix) | svg |
| 62 | G C T A C G T A A G T C A C G T T C G A T A C G A C T G A G C T A G T C T C G A | RORgt(NR)/EL4-RORgt.Flag-ChIP-Seq(GSE56019)/Homer | 1e-60 | -1.395e+02 | 0.0000 | 1308.0 | 3.90% | 205.3 | 2.41% | motif file (matrix) | svg |
| 63 | G C T A C G T A A G T C A C G T T C G A T A C G A C T G A G C T A G T C T C G A | RORgt(NR)/EL4-RORgt.Flag-ChIP-Seq(GSE56019)/Homer | 1e-60 | -1.395e+02 | 0.0000 | 1308.0 | 3.90% | 205.3 | 2.41% | motif file (matrix) | svg |
| 64 | A C G T A G T C A G T C C G A T A C G T A C G T A C T G A C G T A T G C G A C T A C T G T A C G | Sox21(HMG)/ESC-SOX21-ChIP-Seq(GSE110505)/Homer | 1e-56 | -1.310e+02 | 0.0000 | 15581.0 | 46.50% | 3600.2 | 42.17% | motif file (matrix) | svg |
| 65 | A C T G C A G T A C G T C G T A C G T A A C G T A C T G C T G A | Nkx6.1(Homeobox)/Islet-Nkx6.1-ChIP-Seq(GSE40975)/Homer | 1e-53 | -1.238e+02 | 0.0000 | 22272.0 | 66.47% | 5326.6 | 62.40% | motif file (matrix) | svg |
| 66 | C G T A C G T A C G T A A G T C A G C T C T G A A C T G C T A G A G C T A G T C C G T A T C A G | RORg(NR)/Liver-Rorc-ChIP-Seq(GSE101115)/Homer | 1e-51 | -1.191e+02 | 0.0000 | 1041.0 | 3.11% | 160.7 | 1.88% | motif file (matrix) | svg |
| 67 | T G A C G T A C C G T A A C T G T G A C C G A T A C T G A T C G A G C T T A C G T C G A T A G C G T A C C G T A A T C G T G A C G C A T A C T G A C T G A T G C | Twist(bHLH)/HMLE-TWIST1-ChIP-Seq(Chang\_et\_al)/Homer | 1e-50 | -1.170e+02 | 0.0000 | 1809.0 | 5.40% | 319.3 | 3.74% | motif file (matrix) | svg |
| 68 | T A C G T C G A C A G T A C T G G C T A A T G C C G A T G T A C C G T A A C T G T A G C C G T A | NF-E2(bZIP)/K562-NFE2-ChIP-Seq(GSE31477)/Homer | 1e-49 | -1.133e+02 | 0.0000 | 798.0 | 2.38% | 116.0 | 1.36% | motif file (matrix) | svg |
| 69 | C G T A C G T A C G T A G C A T G C A T T A C G G T A C G A C T A C T G C G T A A T C G A C G T G T A C C G T A A G C T | Bach1(bZIP)/K562-Bach1-ChIP-Seq(GSE31477)/Homer | 1e-44 | -1.034e+02 | 0.0000 | 817.0 | 2.44% | 123.0 | 1.44% | motif file (matrix) | svg |
| 70 | T C A G T A G C G A C T C A T G C T G A A T C G G C A T G T A C C G T A A C T G T A G C T G C A | MafK(bZIP)/C2C12-MafK-ChIP-Seq(GSE36030)/Homer | 1e-41 | -9.575e+01 | 0.0000 | 2322.0 | 6.93% | 444.9 | 5.21% | motif file (matrix) | svg |
| 71 | C T G A T C G A C G T A A T G C C G T A C G T A C G A T C T A G T C A G G A T C | Sox15(HMG)/CPA-Sox15-ChIP-Seq(GSE62909)/Homer | 1e-40 | -9.291e+01 | 0.0000 | 10043.0 | 29.97% | 2279.8 | 26.71% | motif file (matrix) | svg |
| 72 | A G T C C G A T C T G A C G T A A C G T C A G T T C A G T G A C | Isl1(Homeobox)/Neuron-Isl1-ChIP-Seq(GSE31456)/Homer | 1e-39 | -9.196e+01 | 0.0000 | 17285.0 | 51.58% | 4093.9 | 47.96% | motif file (matrix) | svg |
| 73 | G T C A G C A T A C T G G T A C G A C T A C T G G C T A A T C G C A G T G T A C C G T A A G C T | Nrf2(bZIP)/Lymphoblast-Nrf2-ChIP-Seq(GSE37589)/Homer | 1e-38 | -8.853e+01 | 0.0000 | 650.0 | 1.94% | 95.4 | 1.12% | motif file (matrix) | svg |
| 74 | G C A T C G T A C G A T A C T G A G T C G C T A C T G A C G T A C A G T A C T G C G T A T C A G | Oct6(POU,Homeobox)/NPC-Pou3f1-ChIP-Seq(GSE35496)/Homer | 1e-37 | -8.702e+01 | 0.0000 | 5805.0 | 17.32% | 1260.5 | 14.77% | motif file (matrix) | svg |
| 75 | A C T G G T C A A C G T G C A T C G A T T C A G G T A C G T C A A C G T C T G A | Oct11(POU,Homeobox)/NCIH1048-POU2F3-ChIP-seq(GSE115123)/Homer | 1e-34 | -8.045e+01 | 0.0000 | 4557.0 | 13.60% | 972.1 | 11.39% | motif file (matrix) | svg |
| 76 | G A C T C G T A A C G T A C T G A G T C C G T A C T G A C G T A C A G T A C G T G T C A T C A G | Brn1(POU,Homeobox)/NPC-Brn1-ChIP-Seq(GSE35496)/Homer | 1e-34 | -8.003e+01 | 0.0000 | 4487.0 | 13.39% | 956.7 | 11.21% | motif file (matrix) | svg |
| 77 | C G T A C G T A C G T A G C A T G C A T A C T G G T A C G A C T C T A G G C T A T A C G G A C T T G A C C G T A A G C T | NFE2L2(bZIP)/HepG2-NFE2L2-ChIP-Seq(Encode)/Homer | 1e-34 | -7.874e+01 | 0.0000 | 888.0 | 2.65% | 146.8 | 1.72% | motif file (matrix) | svg |
| 78 | A T G C G A T C C G A T A C G T A C G T A C T G C A G T A G C T | Sox3(HMG)/NPC-Sox3-ChIP-Seq(GSE33059)/Homer | 1e-32 | -7.537e+01 | 0.0000 | 15169.0 | 45.27% | 3587.7 | 42.03% | motif file (matrix) | svg |
| 79 | T G A C T A G C T C A G T C G A T C G A C G T A A G T C C G T A C G T A C G A T C T A G T A C G | Sox7(HMG)/ESC-Sox7-ChIP-Seq(GSE133899)/Homer | 1e-31 | -7.161e+01 | 0.0000 | 2695.0 | 8.04% | 548.6 | 6.43% | motif file (matrix) | svg |
| 80 | T A G C C T A G T C G A G A C T A C T G C T G A A G T C T C A G G C A T T G A C C T G A A G C T | Atf7(bZIP)/3T3L1-Atf7-ChIP-Seq(GSE56872)/Homer | 1e-28 | -6.549e+01 | 0.0000 | 3939.0 | 11.76% | 844.9 | 9.90% | motif file (matrix) | svg |
| 81 | C G T A A C G T A G C T C G A T C T A G G T A C C G T A A G C T C G T A G C T A | Oct4(POU,Homeobox)/mES-Oct4-ChIP-Seq(GSE11431)/Homer | 1e-26 | -6.040e+01 | 0.0000 | 6328.0 | 18.88% | 1423.7 | 16.68% | motif file (matrix) | svg |
| 82 | C G T A C G T A C G T A A G T C A G C T C T G A A C T G A C T G A G C T A G T C C G T A C T A G C T A G C T A G T G C A | RORa(NR)/Liver-Rora-ChIP-Seq(GSE101115)/Homer | 1e-25 | -5.765e+01 | 0.0000 | 1456.0 | 4.35% | 280.3 | 3.28% | motif file (matrix) | svg |
| 83 | A T G C G A T C C G T A A G C T C A G T A T C G G C A T A G C T G A C T A C T G | Sox17(HMG)/Endoderm-Sox17-ChIP-Seq(GSE61475)/Homer | 1e-24 | -5.699e+01 | 0.0000 | 6840.0 | 20.41% | 1553.1 | 18.19% | motif file (matrix) | svg |
| 84 | T A G C T A G C G A C T C T A G A G C T A G T C G T C A T G C A A C G T A T G C G C T A T G C A | Pbx3(Homeobox)/GM12878-PBX3-ChIP-Seq(GSE32465)/Homer | 1e-24 | -5.639e+01 | 0.0000 | 2326.0 | 6.94% | 479.0 | 5.61% | motif file (matrix) | svg |
| 85 | G A C T C G A T C T G A G T C A G A C T C G A T T C G A C G T A G C T A G C T A T G A C G T A C C G T A A C T G T G C A C G A T A C T G A C G T | Pitx1:Ebox(Homeobox,bHLH)/Hindlimb-Pitx1-ChIP-Seq(GSE41591)/Homer | 1e-24 | -5.551e+01 | 0.0000 | 3395.0 | 10.13% | 728.8 | 8.54% | motif file (matrix) | svg |
| 86 | G T A C G A C T C G T A C T G A T C G A C G T A G C T A C A G T C T G A T A C G | Mef2a(MADS)/HL1-Mef2a.biotin-ChIP-Seq(GSE21529)/Homer | 1e-23 | -5.406e+01 | 0.0000 | 6971.0 | 20.80% | 1590.9 | 18.64% | motif file (matrix) | svg |
| 87 | A G T C A G T C C G A T A C G T A C G T A C T G A C G T A G C T A G T C A G T C | Sox4(HMG)/proB-Sox4-ChIP-Seq(GSE50066)/Homer | 1e-21 | -4.957e+01 | 0.0000 | 7774.0 | 23.20% | 1795.4 | 21.03% | motif file (matrix) | svg |
| 88 | C A T G A C T G C T A G T C G A T C G A T C G A T C G A T C A G T C A G T C A G T G A C T G A C C G T A A C T G T G C A C G A T A C T G | RBPJ:Ebox(?,bHLH)/Panc1-Rbpj1-ChIP-Seq(GSE47459)/Homer | 1e-20 | -4.690e+01 | 0.0000 | 3643.0 | 10.87% | 798.4 | 9.35% | motif file (matrix) | svg |
| 89 | C T G A C T A G A T C G A G C T A C T G G A C T A G T C C T G A | Tbx5(T-box)/HL1-Tbx5.biotin-ChIP-Seq(GSE21529)/Homer | 1e-19 | -4.468e+01 | 0.0000 | 23904.0 | 71.34% | 5894.9 | 69.05% | motif file (matrix) | svg |
| 90 | A T G C A G T C G C A T A G C T A C G T T C A G C G A T A G C T G A T C A T C G | Sox10(HMG)/SciaticNerve-Sox3-ChIP-Seq(GSE35132)/Homer | 1e-18 | -4.264e+01 | 0.0000 | 14333.0 | 42.77% | 3447.0 | 40.38% | motif file (matrix) | svg |
| 91 | T A C G A T G C G A C T A C T G A G C T A G T C G T C A T G C A A C G T A G T C G C T A T G C A | Pknox1(Homeobox)/ES-Prep1-ChIP-Seq(GSE63282)/Homer | 1e-18 | -4.242e+01 | 0.0000 | 2098.0 | 6.26% | 440.6 | 5.16% | motif file (matrix) | svg |
| 92 | C T A G T A C G G A T C G T A C G C T A A G C T A G C T G T C A T C G A T A G C | Nanog(Homeobox)/mES-Nanog-ChIP-Seq(GSE11724)/Homer | 1e-17 | -4.090e+01 | 0.0000 | 26983.0 | 80.52% | 6709.1 | 78.59% | motif file (matrix) | svg |
| 93 | A T G C T C A G T C G A G C A T A C T G C G T A A G T C T C A G G A C T T G A C C G T A A G C T | Atf2(bZIP)/3T3L1-Atf2-ChIP-Seq(GSE56872)/Homer | 1e-16 | -3.882e+01 | 0.0000 | 2698.0 | 8.05% | 585.7 | 6.86% | motif file (matrix) | svg |
| 94 | A T G C C T G A A T C G T A C G A G T C C G A T T C A G C G A T C T A G A G C T G T C A G T C A C G T A A G T C C G T A T A C G C T G A | Fox:Ebox(Forkhead,bHLH)/Panc1-Foxa2-ChIP-Seq(GSE47459)/Homer | 1e-16 | -3.803e+01 | 0.0000 | 10948.0 | 32.67% | 2608.4 | 30.55% | motif file (matrix) | svg |
| 95 | T A C G T C G A G A C T A C T G C T G A A G T C T C A G G A C T T G A C C T G A | Atf1(bZIP)/K562-ATF1-ChIP-Seq(GSE31477)/Homer | 1e-16 | -3.750e+01 | 0.0000 | 5768.0 | 17.21% | 1327.6 | 15.55% | motif file (matrix) | svg |
| 96 | T C G A C T G A C T G A A T G C G T A C G T C A A G C T A G C T C G T A T C A G | Barx1(Homeobox)/Stomach-Barx1.3xFlag-ChIP-Seq(GSE69483)/Homer | 1e-15 | -3.589e+01 | 0.0000 | 5763.0 | 17.20% | 1329.2 | 15.57% | motif file (matrix) | svg |
| 97 | A T G C A G T C G T A C A G C T T C G A C T A G G A T C C T G A G T C A A G T C G C T A T C A G | Rfx5(HTH)/GM12878-Rfx5-ChIP-Seq(GSE31477)/Homer | 1e-14 | -3.446e+01 | 0.0000 | 4062.0 | 12.12% | 918.6 | 10.76% | motif file (matrix) | svg |
| 98 | C A T G A G T C A G C T C G T A C G A T C G A T G C A T G C A T C G A T C T G A C A T G T G A C | Mef2d(MADS)/Retina-Mef2d-ChIP-Seq(GSE61391)/Homer | 1e-14 | -3.418e+01 | 0.0000 | 3915.0 | 11.68% | 883.1 | 10.34% | motif file (matrix) | svg |
| 99 | T C G A A C G T A C T G C T G A A G T C T C A G A G C T G T A C C G T A A G C T G A T C T C G A | JunD(bZIP)/K562-JunD-ChIP-Seq/Homer | 1e-14 | -3.241e+01 | 0.0000 | 792.0 | 2.36% | 152.0 | 1.78% | motif file (matrix) | svg |
| 100 | T C G A A C T G A C T G C G T A C G T A T C G A A G T C C T G A A T C G G T A C G C A T C A T G | ETS:E-box(ETS,bHLH)/HPC7-Scl-ChIP-Seq(GSE22178)/Homer | 1e-13 | -3.157e+01 | 0.0000 | 1259.0 | 3.76% | 258.8 | 3.03% | motif file (matrix) | svg |
| 101 | C G T A C T A G G A C T G T C A G T C A C G T A A G T C C G T A T C G A T C G A T C G A C G T A C T G A C T A G G C T A C G T A T A G C C G T A C G A T C G T A | FOXA1:AR(Forkhead,NR)/LNCAP-AR-ChIP-Seq(GSE27824)/Homer | 1e-12 | -2.837e+01 | 0.0000 | 938.0 | 2.80% | 188.3 | 2.21% | motif file (matrix) | svg |
| 102 | G C T A A C G T C T A G G T A C G C T A G A C T C T G A G C A T C A T G G A T C | Pit1(Homeobox)/GCrat-Pit1-ChIP-Seq(GSE58009)/Homer | 1e-11 | -2.602e+01 | 0.0000 | 12410.0 | 37.03% | 3009.8 | 35.26% | motif file (matrix) | svg |
| 103 | C T A G T A G C A G T C G T C A C T G A A C G T C G T A C G T A C G T A G C T A | Hoxd13(Homeobox)/ChickenMSG-Hoxd13.Flag-ChIP-Seq(GSE86088)/Homer | 1e-11 | -2.579e+01 | 0.0000 | 16892.0 | 50.41% | 4145.6 | 48.56% | motif file (matrix) | svg |
| 104 | T C G A G C A T A C T G C T G A A G T C T C A G G A C T G T A C C G T A A G C T A G T C G A T C | c-Jun-CRE(bZIP)/K562-cJun-ChIP-Seq(GSE31477)/Homer | 1e-11 | -2.565e+01 | 0.0000 | 2553.0 | 7.62% | 570.4 | 6.68% | motif file (matrix) | svg |
| 105 | C T G A C G A T C T A G T C A G G A T C C T G A T C A G G A T C C T G A A C T G A G T C G C T A A C G T A G T C G C A T | PRDM9(Zf)/Testis-DMC1-ChIP-Seq(GSE35498)/Homer | 1e-10 | -2.400e+01 | 0.0000 | 4159.0 | 12.41% | 962.5 | 11.27% | motif file (matrix) | svg |
| 106 | T G A C G C T A T G A C C G T A T C A G G A T C C G T A C A T G C A T G C T A G C T A G C T A G | Unknown-ESC-element(?)/mES-Nanog-ChIP-Seq(GSE11724)/Homer | 1e-10 | -2.353e+01 | 0.0000 | 4800.0 | 14.32% | 1120.5 | 13.13% | motif file (matrix) | svg |
| 107 | G T C A G C A T G C T A C A G T C T A G G A T C C G T A C T G A C G T A C G A T | Oct2(POU,Homeobox)/Bcell-Oct2-ChIP-Seq(GSE21512)/Homer | 1e-10 | -2.307e+01 | 0.0000 | 3950.0 | 11.79% | 913.1 | 10.70% | motif file (matrix) | svg |
| 108 | C G A T A C G T C G T A C G T A A C G T A G C T T G C A C T G A C G T A A C G T A C G T C G T A | Phox2b(Homeobox)/CLBGA-PHOX2B-ChIP-Seq(GSE90683)/Homer | 1e-9 | -2.186e+01 | 0.0000 | 2559.0 | 7.64% | 578.6 | 6.78% | motif file (matrix) | svg |
| 109 | G C T A A C G T C T A G G T C A C G T A A C G T C G T A C G A T C A G T A G T C C G T A A C G T C T A G C T G A A T C G | OCT:OCT(POU,Homeobox)/NPC-Brn1-ChIP-Seq(GSE35496)/Homer | 1e-9 | -2.132e+01 | 0.0000 | 190.0 | 0.57% | 30.2 | 0.35% | motif file (matrix) | svg |
| 110 | T G C A T C G A T C G A A C G T C A T G C G T A A G T C T C A G A C G T G T A C C G T A A G C T | CREB5(bZIP)/LNCaP-CREB5.V5-ChIP-Seq(GSE137775)/Homer | 1e-9 | -2.121e+01 | 0.0000 | 3933.0 | 11.74% | 913.7 | 10.70% | motif file (matrix) | svg |
| 111 | G C T A C G A T C T A G G T A C G C T A C G A T C G T A G C T A C G A T C A G T G A T C G C T A | Pit1+1bp(Homeobox)/GCrat-Pit1-ChIP-Seq(GSE58009)/Homer | 1e-8 | -1.886e+01 | 0.0000 | 4720.0 | 14.09% | 1112.6 | 13.03% | motif file (matrix) | svg |
| 112 | G A C T G A C T T C G A C G T A C G A T A G C T C T G A A C T G T G A C G A C T T C G A C G T A A C G T A G C T C T G A C T G A G T C A G C T A G C T A C G T A | Pax7(Paired,Homeobox),longest/Myoblast-Pax7-ChIP-Seq(GSE25064)/Homer | 1e-7 | -1.813e+01 | 0.0000 | 381.0 | 1.14% | 72.3 | 0.85% | motif file (matrix) | svg |
| 113 | C T A G A G C T G A C T C A T G A G T C A G T C G T C A C A G T C T A G T C A G G T A C C T G A T C G A G A T C T G A C | Rfx2(HTH)/LoVo-RFX2-ChIP-Seq(GSE49402)/Homer | 1e-7 | -1.768e+01 | 0.0000 | 1624.0 | 4.85% | 361.5 | 4.23% | motif file (matrix) | svg |
| 114 | C G T A G A C T C G A T A T C G G T A C G C A T C A T G C G T A T A C G G C A T G T A C C G T A C A T G A T G C G C T A C T A G G C A T G C A T G C A T G A C T | MafB(bZIP)/BMM-Mafb-ChIP-Seq(GSE75722)/Homer | 1e-6 | -1.552e+01 | 0.0000 | 4484.0 | 13.38% | 1063.8 | 12.46% | motif file (matrix) | svg |
| 115 | C T A G C T A G A C T G A G C T G A C T A G C T A C G T C T A G G T C A C T G A A C T G T C A G C T G A A C G T C T A G G C T A C T G A A C G T C G T A C T A G A C T G C G T A A C T G A G C T A C G T | ZFP3(Zf)/HEK293-ZFP3.GFP-ChIP-Seq(GSE58341)/Homer | 1e-6 | -1.548e+01 | 0.0000 | 18.0 | 0.05% | 0.0 | 0.00% | motif file (matrix) | svg |
| 116 | G C A T C T G A T G C A G A C T A G T C G T A C T C A G C G T A A G C T A C G T G C T A G T A C | Pax7(Paired,Homeobox),long/Myoblast-Pax7-ChIP-Seq(GSE25064)/Homer | 1e-6 | -1.463e+01 | 0.0000 | 324.0 | 0.97% | 62.9 | 0.74% | motif file (matrix) | svg |
| 117 | C G A T T A C G T G C A G T A C G A T C G A C T A G C T A C G T A T C G G T A C G A T C G T A C G A T C G T C A | PPARE(NR),DR1/3T3L1-Pparg-ChIP-Seq(GSE13511)/Homer | 1e-6 | -1.418e+01 | 0.0000 | 6071.0 | 18.12% | 1461.3 | 17.12% | motif file (matrix) | svg |
| 118 | A C G T T A C G G A T C A C T G A C G T C T A G A C T G A C T G G A T C C T A G C A T G C T A G | Egr2(Zf)/Thymocytes-Egr2-ChIP-Seq(GSE34254)/Homer | 1e-5 | -1.294e+01 | 0.0000 | 1302.0 | 3.89% | 292.3 | 3.42% | motif file (matrix) | svg |
| 119 | C T A G C A T G C A T G A G C T G A C T C G T A G A T C A G C T G T C A A T G C T C G A C T A G C A T G A C G T A G T C C T G A | LXRE(NR),DR4/RAW-LXRb.biotin-ChIP-Seq(GSE21512)/Homer | 1e-5 | -1.275e+01 | 0.0000 | 304.0 | 0.91% | 59.4 | 0.70% | motif file (matrix) | svg |
| 120 | T G A C C G T A C T G A A C T G A C T G G A C T G A T C T G C A G T A C T A C G | SF1(NR)/H295R-Nr5a1-ChIP-Seq(GSE44220)/Homer | 1e-4 | -1.136e+01 | 0.0000 | 3223.0 | 9.62% | 764.1 | 8.95% | motif file (matrix) | svg |
| 121 | T A C G A T C G G A T C G T C A C T G A G C A T C G A T G C T A T C G A G C T A | Unknown(Homeobox)/Limb-p300-ChIP-Seq/Homer | 1e-4 | -1.122e+01 | 0.0000 | 7625.0 | 22.76% | 1861.3 | 21.80% | motif file (matrix) | svg |
| 122 | G C T A A G C T T A C G G T C A C T G A C G A T C T G A G C A T C A G T A G T C | Brn2(POU,Homeobox)/NPC-Brn2-ChIP-Seq(GSE35496)/Homer | 1e-4 | -1.113e+01 | 0.0001 | 1508.0 | 4.50% | 345.9 | 4.05% | motif file (matrix) | svg |
| 123 | T A G C A G T C T G A C A G T C C T A G A T C G A G T C C A T G T G A C A G T C G T A C A G T C A G T C G C A T C T A G A T C G G C A T A C T G A T C G G A T C | BORIS(Zf)/K562-CTCFL-ChIP-Seq(GSE32465)/Homer | 1e-4 | -1.074e+01 | 0.0001 | 834.0 | 2.49% | 184.8 | 2.16% | motif file (matrix) | svg |
| 124 | A C T G T G A C G T A C C G T A A G T C T A C G A C G T A C T G G T C A A G T C | NPAS2(bHLH)/Liver-NPAS2-ChIP-Seq(GSE39860)/Homer | 1e-4 | -1.028e+01 | 0.0001 | 9457.0 | 28.22% | 2326.2 | 27.25% | motif file (matrix) | svg |
| 125 | C G T A T A G C A G T C C T A G C A G T C T A G C T G A G T A C G C A T T C G A C G T A G C A T A G C T C T A G T C G A | PAX3:FKHR-fusion(Paired,Homeobox)/Rh4-PAX3:FKHR-ChIP-Seq(GSE19063)/Homer | 1e-4 | -1.017e+01 | 0.0001 | 2372.0 | 7.08% | 558.9 | 6.55% | motif file (matrix) | svg |
| 126 | C G A T C T A G T C G A A G C T C G A T C T G A C G T A A G C T A C T G C T A G A T G C G A T C | Hoxb4(Homeobox)/ES-Hoxb4-ChIP-Seq(GSE34014)/Homer | 1e-4 | -1.005e+01 | 0.0002 | 1983.0 | 5.92% | 463.6 | 5.43% | motif file (matrix) | svg |
| 127 | C T A G T A C G G A T C G T A C C T G A A G C T T G C A G C T A C G T A G C A T G A T C G C T A | Hoxc9(Homeobox)/Ainv15-Hoxc9-ChIP-Seq(GSE21812)/Homer | 1e-3 | -9.102e+00 | 0.0004 | 5708.0 | 17.03% | 1390.4 | 16.29% | motif file (matrix) | svg |
| 128 | A C T G C T A G G A T C C G T A C T A G A G T C T G C A G A C T C G T A A C G T T C A G A G T C A C G T C T G A A G T C A G T C G A T C C G T A T C A G T A C G | EBNA1(EBV-virus)/Raji-EBNA1-ChIP-Seq(GSE30709)/Homer | 1e-3 | -9.049e+00 | 0.0004 | 123.0 | 0.37% | 22.8 | 0.27% | motif file (matrix) | svg |
| 129 | A G T C A T C G G C A T C T A G A C T G T A C G C G A T T C A G C A T G A G C T T A G C G A T C | GLI3(Zf)/Limb-GLI3-ChIP-Chip(GSE11077)/Homer | 1e-3 | -9.005e+00 | 0.0004 | 730.0 | 2.18% | 162.4 | 1.90% | motif file (matrix) | svg |
| 130 | G A C T C T A G G A T C C A G T A C T G C T G A A T G C G C A T A T G C C T G A | MafA(bZIP)/Islet-MafA-ChIP-Seq(GSE30298)/Homer | 1e-3 | -8.997e+00 | 0.0004 | 7433.0 | 22.18% | 1823.7 | 21.36% | motif file (matrix) | svg |
| 131 | C A T G T A C G T A G C G A T C G A T C A T G C G T A C G A C T T C A G A T G C C G A T A T C G C A G T A C T G G T A C | Zic3(Zf)/mES-Zic3-ChIP-Seq(GSE37889)/Homer | 1e-3 | -8.402e+00 | 0.0008 | 3865.0 | 11.53% | 933.3 | 10.93% | motif file (matrix) | svg |
| 132 | A T C G A G C T A C T G A G T C A C T G A G T C C G T A A C G T A C T G A G T C A C T G A G T C | NRF(NRF)/Promoter/Homer | 1e-3 | -8.335e+00 | 0.0008 | 784.0 | 2.34% | 176.9 | 2.07% | motif file (matrix) | svg |
| 133 | C G T A C T A G T C A G T C A G A G T C A T G C A G T C G C A T A G C T A C G T A T C G C G A T | Sox9(HMG)/Limb-SOX9-ChIP-Seq(GSE73225)/Homer | 1e-3 | -8.295e+00 | 0.0008 | 7916.0 | 23.62% | 1948.2 | 22.82% | motif file (matrix) | svg |
| 134 | A C T G T C A G A G C T G A C T C A T G A G T C A G T C G C T A C G A T C T A G T C A G G T A C C T G A T C G A | Rfx1(HTH)/NPC-H3K4me1-ChIP-Seq(GSE16256)/Homer | 1e-3 | -7.975e+00 | 0.0011 | 2678.0 | 7.99% | 640.9 | 7.51% | motif file (matrix) | svg |
| 135 | G C T A G C A T G A C T G C A T T C A G G T A C G C T A G C A T C T G A G C T A T A G C G C T A C T G A C G A T C T A G | OCT4-SOX2-TCF-NANOG(POU,Homeobox,HMG)/mES-Oct4-ChIP-Seq(GSE11431)/Homer | 1e-3 | -7.949e+00 | 0.0012 | 2617.0 | 7.81% | 625.9 | 7.33% | motif file (matrix) | svg |
| 136 | A G T C G T A C A G C T C T A G A G T C C G A T A C T G C G T A A C T G G T C A | Zic(Zf)/Cerebellum-ZIC1.2-ChIP-Seq(GSE60731)/Homer | 1e-3 | -6.957e+00 | 0.0031 | 6882.0 | 20.54% | 1695.1 | 19.86% | motif file (matrix) | svg |
| 137 | C T G A T C A G A C T G C A G T A G T C G A C T A G T C A C G T C G T A G C T A A T G C G A T C | PRDM14(Zf)/H1-PRDM14-ChIP-Seq(GSE22767)/Homer | 1e-3 | -6.952e+00 | 0.0031 | 2579.0 | 7.70% | 619.7 | 7.26% | motif file (matrix) | svg |
| 138 | T A G C G A T C G T A C C T G A G C A T C T G A C G T A T G C A C G T A G A T C | Hoxa13(Homeobox)/ChickenMSG-Hoxa13.Flag-ChIP-Seq(GSE86088)/Homer | 1e-2 | -6.715e+00 | 0.0039 | 22696.0 | 67.73% | 5715.2 | 66.95% | motif file (matrix) | svg |
| 139 | G T A C G C A T C T G A C G T A G A C T A G C T C A T G T G C A C T G A A C G T G A C T C G T A | Prop1(Homeobox)/GHFT1-PROP1.biotin-ChIP-Seq(GSE77302)/Homer | 1e-2 | -6.686e+00 | 0.0040 | 7583.0 | 22.63% | 1873.6 | 21.95% | motif file (matrix) | svg |
| 140 | C T A G A C G T G C A T T C G A G T C A G C A T A T C G C G T A C A G T A G C T C T G A T G C A | HNF1b(Homeobox)/PDAC-HNF1B-ChIP-Seq(GSE64557)/Homer | 1e-2 | -6.675e+00 | 0.0040 | 1579.0 | 4.71% | 373.7 | 4.38% | motif file (matrix) | svg |
| 141 | T A C G T C A G T G C A A G C T T G A C A G C T A G T C A C T G G A T C A C T G T C G A A C T G C T G A C T G A A T G C | ZBTB33(Zf)/GM12878-ZBTB33-ChIP-Seq(GSE32465)/Homer | 1e-2 | -6.632e+00 | 0.0041 | 45.0 | 0.13% | 7.9 | 0.09% | motif file (matrix) | svg |
| 142 | G A C T A G T C C G A T A C T G C T G A T G A C G T A C C G T A A T C G G C A T C T G A C T A G | Bcl11a(Zf)/HSPC-BCL11A-ChIP-Seq(GSE104676)/Homer | 1e-2 | -6.442e+00 | 0.0049 | 4804.0 | 14.34% | 1176.6 | 13.78% | motif file (matrix) | svg |
| 143 | G A T C G C A T C T G A C G T A A G C T A G C T C G T A T C G A C T G A A C G T G C A T C G T A | Phox2a(Homeobox)/Neuron-Phox2a-ChIP-Seq(GSE31456)/Homer | 1e-2 | -6.095e+00 | 0.0069 | 4821.0 | 14.39% | 1182.7 | 13.85% | motif file (matrix) | svg |
| 144 | G T C A A C G T C T A G G T C A C G T A G C A T C G T A C G T A G C A T C A G T A G T C C G T A C A G T C T A G C T G A | OCT:OCT(POU,Homeobox,IR1)/NPC-Brn2-ChIP-Seq(GSE35496)/Homer | 1e-2 | -6.062e+00 | 0.0071 | 289.0 | 0.86% | 62.3 | 0.73% | motif file (matrix) | svg |
| 145 | C T A G A C G T A G T C C G T A A C T G A G T C G C A T A C T G G C A T A G T C G A C T G A T C G C A T A G T C A G C T | ZNF317(Zf)/HEK293-ZNF317.GFP-ChIP-Seq(GSE58341)/Homer | 1e-2 | -5.743e+00 | 0.0097 | 858.0 | 2.56% | 199.0 | 2.33% | motif file (matrix) | svg |
| 146 | T G C A T G C A A G T C A G T C G A C T C A G T A T G C G A T C C T G A A C G T C T A G C T A G A G T C A C G T A G T C A G T C A G T C G A C T C G T A A C G T A G C T C T A G G A T C G A T C G A T C | ZNF16(Zf)/HEK293-ZNF16.GFP-ChIP-Seq(GSE58341)/Homer | 1e-2 | -5.725e+00 | 0.0098 | 53.0 | 0.16% | 9.4 | 0.11% | motif file (matrix) | svg |
| 147 | C T A G A T C G G A C T A G T C A G T C A G T C G A C T C T G A A C T G C T A G A C T G C T G A | EBF(EBF)/proBcell-EBF-ChIP-Seq(GSE21978)/Homer | 1e-2 | -5.638e+00 | 0.0107 | 1400.0 | 4.18% | 332.1 | 3.89% | motif file (matrix) | svg |
| 148 | C T A G A T C G G T C A C A T G A G T C G A C T T A C G C A G T A G T C A G T C C T G A C G A T C T A G A T C G G A C T A T C G A G T C G A C T C T A G T C G A | REST-NRSF(Zf)/Jurkat-NRSF-ChIP-Seq/Homer | 1e-2 | -5.593e+00 | 0.0111 | 38.0 | 0.11% | 6.8 | 0.08% | motif file (matrix) | svg |
| 149 | C T A G A T G C A T G C C G A T A C T G G A C T A T G C G C T A T G A C A G C T T A G C G C T A | PBX1(Homeobox)/MCF7-PBX1-ChIP-Seq(GSE28007)/Homer | 1e-2 | -5.374e+00 | 0.0137 | 760.0 | 2.27% | 176.9 | 2.07% | motif file (matrix) | svg |
| 150 | A G T C A T C G C T A G A G C T G A C T C T A G A G T C A G T C G C T A C A G T T C A G T C A G G A T C C T G A T C G A G A T C | RFX(HTH)/K562-RFX3-ChIP-Seq(SRA012198)/Homer | 1e-2 | -5.031e+00 | 0.0192 | 1437.0 | 4.29% | 343.5 | 4.02% | motif file (matrix) | svg |
| 151 | T C A G T A C G G A T C G T A C T C G A C G A T C T G A C G T A G C T A C G T A | Hoxd11(Homeobox)/ChickenMSG-Hoxd11.Flag-ChIP-Seq(GSE86088)/Homer | 1e-2 | -5.018e+00 | 0.0193 | 21382.0 | 63.81% | 5391.9 | 63.16% | motif file (matrix) | svg |
| 152 | C T G A A T C G A G C T A G C T A C G T T A G C C T G A T A C G C G A T A C G T G A C T A G T C | ISRE(IRF)/ThioMac-LPS-Expression(GSE23622)/Homer | 1e-2 | -4.989e+00 | 0.0197 | 633.0 | 1.89% | 146.4 | 1.72% | motif file (matrix) | svg |
| 153 | T C A G T G A C G T A C T G C A G T A C C T A G G T A C A T G C A G T C G T C A A G T C G A C T | Klf9(Zf)/GBM-Klf9-ChIP-Seq(GSE62211)/Homer | 1e-2 | -4.806e+00 | 0.0235 | 2031.0 | 6.06% | 491.4 | 5.76% | motif file (matrix) | svg |
